# Examining Postoperative Posterior Cruciate Ligament Index: A Structural Paradigm in Anterior Cruciate Ligament (ACL) Reconstructions With Hamstring Tendon Autograft

**DOI:** 10.7759/cureus.53089

**Published:** 2024-01-28

**Authors:** Mustafa Yalın, Sefa Key, Mustafa Yıldırım, Anıl Agar

**Affiliations:** 1 Department of Orthopedics and Traumatology, Elazığ Fethi Sekin City Hospital, Elazığ, TUR; 2 Department of Orthopedics and Traumatology, Fırat University, Elazığ, TUR; 3 Department of Radiology, Fırat University, Elazığ, TUR

**Keywords:** arthroscopy, knee, posterior cruciate ligament index, hamstring tendon, anterior cruciate ligament reconstruction

## Abstract

Background: The current study aimed to compare the posterior cruciate ligament (PCL) index values of patients who underwent hamstring tendon (HT) autograft reconstruction due to an anterior cruciate ligament (ACL) tear. The comparison involved assessing these values in a similar cohort and evaluating the association between the alteration in the PCL index and functional results.

Methods: Patients who were clinically diagnosed with a complete, unilateral ACL tear and underwent ACL reconstruction (ACLR) using HT autograft between January 2018 and January 2021 constituted the operated group (Group 1) of the study. The control group (Group 2) consisted of patients selected from a convenience sample without ACL rupture, meniscal lesion, or cartilage damage who underwent an MRI during an outpatient orthopaedic consultation for knee pain. The operated group was submitted for an MRI of the knee one year after the operation for any reason such as pain, graft healing, the presence of tunnel widening, or suspicion of re-rupture. The International Knee Documentation Committee (IKDC) Subjective Knee Evaluation Form and the Lysholm Scoring System were applied to the patients in the operated group in the preoperative and postoperative periods to evaluate their complaints, function, and participation in sports and to assess functional ability and functional capacity. A radiologist with five years of experience measured the PCL index in the sagittal section of an MRI. In the operated group, changes in PCL index, IKDC, and Lysholm values during the postoperative period were assessed, along with their correlation. Additionally, a comparison was made between the values of the operated group and the non-operated group.

Results: No statistically significant correlation was found between the PCL index alteration and the functional score alteration (IKDC and Lysholm) in the operated group (p>0.05). In comparison to the non-operated group, the preoperative PCL index measures of the operated group were significantly lower (p: 0.000; p<0.05). The increase in the postoperative PCL index measurements of the operated group was similarly statistically significant (p: 0.000; p<0.05).

Conclusion: Although the PCL index appears to be a strong anatomical structural parameter in ACLR patients performing HT autograft in the postoperative period, its correlation with functional results is weak.

## Introduction

Knee joint trauma, one of the most reported musculoskeletal injuries, impacts approximately 1080 individuals per 100,000 [1]. Contrary to the high frequency of knee injuries, anterior cruciate ligament (ACL) injuries are relatively rare [2-4]. The diagnosis is suspected based on clinical history; however, it is frequently missed if the rupture is partial [4]. In daily clinical practice, the first diagnostic method for knee joint evaluation is often magnetic resonance imaging (MRI). However, knee arthroscopy serves as the gold standard diagnostic and therapeutic tool due to its exceptional specificity and sensitivity, allowing for the detection of even minor ACL abnormalities [5]. Over 20 direct and indirect indicators of ACL rupture have been identified since the adoption of MRIs in knee exams [5].

The posterior cruciate ligament (PCL) morphology has been utilized as an accompanying indicator of ACL injury. This discovery was investigated subjectively on a sagittal MRI at first [6]. Mink et al. observed that the PCL curves backwards in an ACL-deficient knee, a condition known as "PCL sprain" [6]. Following these results, quantitative measurements for alterations in PCL morphology were developed [5,7,8]. Liu et al. [5] characterized the PCL index as the A/B proportion, where A is the length of the line uniting the PCL's posterior-inferior tibial attachment and superior-anterior femoral attachment and B is the highest vertical length from this line to the PCL. The researchers developed the PCL index to assess the alteration in morphology of the PCL following ACL tears and discovered that ACL deficiency correlated with lower PCL index measurements when compared to knees, including an undamaged ACL [5]. The PCL index has been shown to be sensitive as well as specific in detecting ACL ruptures [5,8]. The PCL index is defined primarily by the impingement of ACL grafts onto the PCL following ACL reconstruction (ACLR) [9]. The PCL index is widely utilized in the field to determine ACLR effectiveness [9]. Nevertheless, there is currently no scientific proof for its clinical significance or association with knee joint biomechanics derived via anatomical ACLR. According to Zampeli et al. [10], the PCL index with anatomical restoration of the ACL demonstrated that tibiofemoral joint configuration correlates directly with improved functional outcomes.

The current study aimed to compare the PCL index values of patients who underwent hamstring tendon (HT) autograft reconstruction owing to ACL tear with those of a control group (a group of patients who presented to the orthopaedic outpatient clinic and underwent MRI for knee pain but were found to be completely healthy) as well as to assess the association between the alteration in the PCL index and the alteration in functional results.

## Materials and methods

After obtaining ethical approval from the Fırat University Faculty of Medicine Local Ethics Committee (approval date: 03/11/2022; approval number: 2022/13-24), a comprehensive assessment was conducted by scanning the hospital records of Elazığ Fethi Sekin City Hospital in Elazığ, Turkey, retrospectively. The operated group (Group 1) of the study consisted of clinically diagnosed male patients with complete ACL rupture, aged younger than 40, clinically unilateral, had ACLR using HT autograft at least two months after the trauma resulting in ACL tear between 2018 and 2021 at a tertiary public hospital in Turkey, had a follow-up period of at least one year after surgery, and had no previous history of ACL injury. The criteria for excluding individuals from the operated group included being over 40 years of age, female gender (outcomes following ACLR vary in both males and females [11], and reproductive hormones impact knee elasticity [12]), individuals who had surgery within two months after experiencing a traumatic event resulting in an ACL injury, patients who did not complete the minimum one-year follow-up period for any reason, and patients who declined to participate in the study. Additionally, individuals with multi-ligament injuries, chondral defects, meniscal rupture, congenital disorders, comorbidities, or prior knee surgery were also excluded. Those individuals with unilateral ACL rupture were submitted for an MRI of the knee one year after the operation for any reason such as pain, graft healing, the presence of tunnel widening, or suspicion of re-rupture. To ensure comparability with Group 1, a control group (Group 2) comprising 96 individuals was constructed. Group 2, serving as the control group in the research, consisted of male patients aged under 40 who visited the orthopaedic outpatient clinic due to knee pain. These patients were confirmed to be in a state of perfect health after undergoing an MRI. The control group was excluded if they had an ACL injury or meniscal injury, had cartilage damage, were outside the specified age range, were female, had prior knee surgery, had a multi-ligament injury, had comorbidities, or had a history of congenital disease. All patients provided the written informed consent form in compliance with the hospital's ethical committee's norms.

MRI examinations were performed using a multichannel 1.5 Tesla (Philips Healthcare, Andover, Massachusetts, United States). The patients' symptoms, performance, and involvement in sports activities were determined using the International Knee Documentation Committee (IKDC) Subjective Knee Evaluation Form [13]. To test functional capacity, the Lysholm Scoring System [14] was utilized. The IKDC (0-100%) and Lysholm (0-100%) scores were assessed both before and after surgical reconstruction (one year), which occurred at the same time as the MRI. Patients completed the IKDC questionnaire individually, while an orthopaedic surgeon (MY, the first author of the study) determined the Lysholm score.

**Figure 1:**
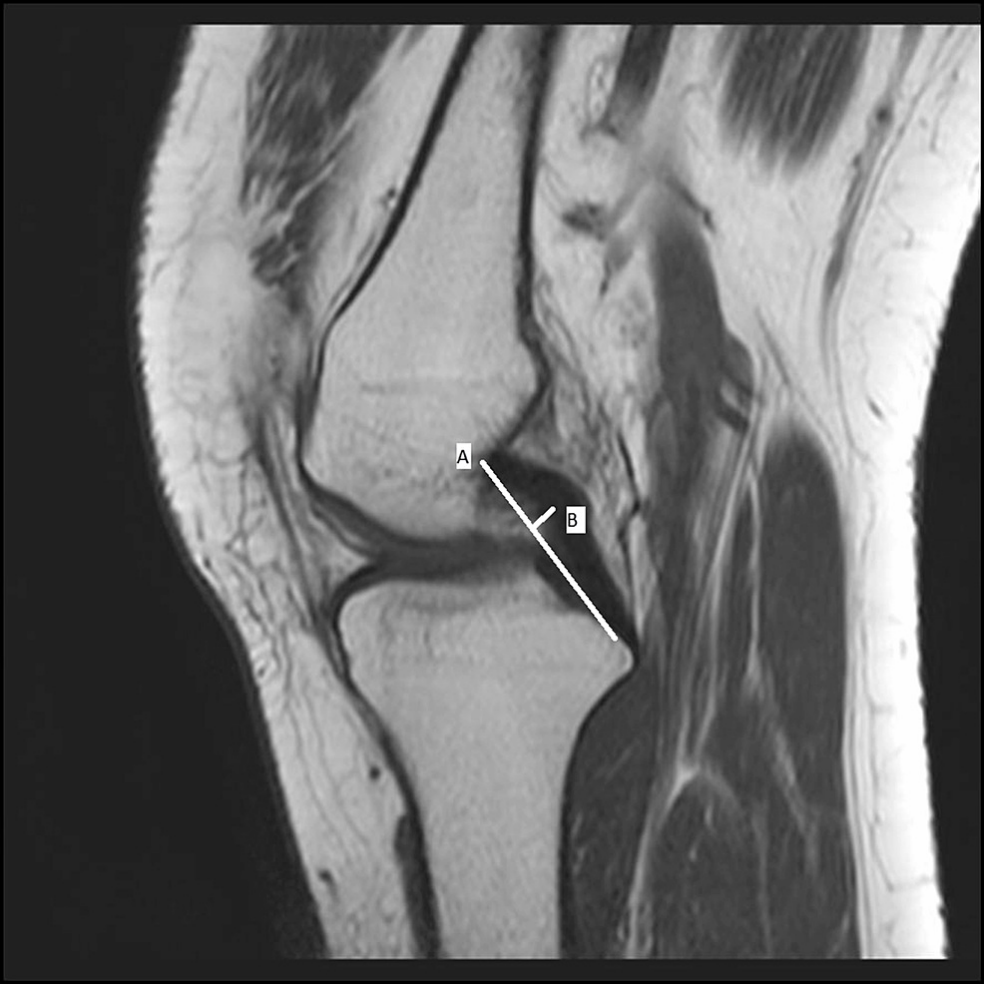
Evaluation of the PCL index. Each measurement was obtained from a single oblique sagittal image that clearly shows the full length of the PCL from its origin in the femur, through the joint space, and into the tibia. The PCL index equals the ratio A/B, where A is the length of the line between the posterior inferior tibial attachment and the superior anterior femoral attachment of the PCL and B is the greatest vertical distance of this line to the PCL. PCL: posterior cruciate ligament

In the sagittal MRI plane, the PCL index was determined by employing the Liu method [5] (Figure 1).

A radiologist with five years of experience (MY, the third author of the study) measured the PCL index. An orthopaedic surgeon with 10 years of experience (AA, the fourth author of the study) gathered and documented all statistics. The participants were separated into two categories: those who suffered ACL rupture (Group 1) and those who did not (Group 2).

Surgical procedure

General or spinal anaesthesia was administered to the patients. They were operated on arthroscopically by a specialist surgical team, and the homolateral HTs (both semitendinosus and gracilis) were applied. The anatomical femoral single-tunnel method was utilized for the reconstruction. The autograft was fixed via an Endobutton® fixation device (Smith & Nephew®, London, United Kingdom) in the femoral tunnel and a bioabsorbable interference screw and a U-shaped staple at the tibial level. Early range of motion as well as isometric and isokinetic exercises were encouraged.

Statistics

For statistical analysis of the study's findings, the IBM SPSS Statistics for Windows, Version 22.0 (Released 2013; IBM Corp., Armonk, New York, United States) application was employed. The Shapiro-Wilk test was utilized to evaluate the parameters' conformance to the normal distribution when assessing the study data. When analysing the study data, the Student's t-test was employed to compare normally distributed parameters between two groups in quantitative and descriptive statistical methods (mean, standard deviation, frequency). For in-group comparisons of normally distributed quantitative data, the paired sample t-test was employed, and for non-normally distributed parameters, the Wilcoxon signed-rank test was utilized. To compare qualitative data, the chi-squared test was applied. Pearson correlation analysis was performed to investigate the correlations between normally distributed parameters. The significance was determined at the p<0.05 level.

## Results

In total, 106 patients were identified; seven patients were excluded due to a concomitant meniscal tear, and four patients were excluded due to an accompanying chondral lesion. The sample consisted of 95 male patients. All of them were active in their daily lives before the ligament injury. The right ACLR was conducted on 47 cases, and the left ACLR was conducted on 48 cases. Table 1 shows the ages and sides of the patients according to the groups.

**Table 1:** Evaluation of the operated side and age according to the state of being operated ^1^Student's t-test; ^2^chi-squared test

		Operated		Non-operated		Total		p
Demographics		Min-max	Mean±SD	Min-max	Mean±SD	Min-max	Mean±SD	
Age (years)		16-40	25.72±6.19	15-39	27.4±5.96	15-40	26.56±6.12	^1^0.058
Side _n(%)_								
Right		47 (49.5%)		50 (52.1%)		97 (50.8%)		^2^0.718
Left		48 (50.5%)		46 (47.9%)		94 (49.2%)		

According to the results of the preoperative Lysholm score, the extent of the change seen in the postoperative values varied between zero and 47; the average was 26.96±11.11, and the median was 28. According to the preoperative IKDC values, the extent of the change seen in the postoperative values varied between four and 46, with an average of 29.23±9.51 and a median of 30 (Table 2).

**Table 2:** Information on the change values observed in the postoperative period compared to the preoperative period in the operated group PCL: posterior cruciate ligament; IKDC: International Knee Documentation Committee

Postop-preop	Operated	
	Min-max	Mean±SD
PCL index change	0.31-4.69	2.16±0.89
Lysholm score change	0-47	26.96±11.11
IKDC change	4-46	29.23±9.51

According to the preoperative PCL index values, the extent of the change seen in the postoperative values varied between 0.31 and 4.69, with an average of 2.16±0.89. The preoperative PCL index amounts of the operated group were statistically significantly lower compared to the non-operated group (p: 0.000; p<0.05). The increase in the postoperative PCL index values of the operated group was also statistically significant (p: 0.000; p<0.05) (Table 3).

**Table 3:** Evaluation of preoperative and postoperative values between groups ^1^Student's t-test; ^2^paired sample t-test; ^3^Wilcoxon signed-rank test; *p<0.05 PCL: posterior cruciate ligament; IKDC: International Knee Documentation Committee

Evaluation parameter		Operated			Non-operated			Total			p^1^
		Min-max	Mean±SD		Min-max	Mean±SD		Min-max	Mean±SD		
PCL index	Preop	3.03-7.75		4.67±0.87	5.86-9.99		7.93±0.96	3.03-9.99		6.31±1.87	0.000*
	Postop	4.78-9.9		6.83±1.14	-			4.78-9.9		6.83±1.14	-
	Preop-postop p^2^	0.000*			-			-			
Lysholm score (0-100)	Preop	45-65		53.37±5.85	-			45-65		53.37±5.85	-
	Postop	55-95		80.33±9.31	-			55-95		80.33±9.31	-
	Preop-postop p^3^	0.000*			-			-			
IKDC (0-100)	Preop	42-66		53.45±5.75	-			42-66		53.45±5.75	-
	Postop	58-96		82.68±8.17	-			58-96		82.68±8.17	-
	Preop-postop p^3^	0.000*			-			-			

When evaluated with Pearson correlation analysis, no statistically significant relationship was found between the extent of the change seen in the postoperative values according to the preoperative PCL index values and the extent of the change seen in the postoperative values according to the preoperative Lysholm and IKDC scores (p>0.05) (Table 4).

**Table 4:** Evaluation of the correlation between the changes observed in the postoperative period compared to the preoperative period in the operated group Pearson correlation analysis; r: the correlation and direction of the relationship between the parameters; p: the significance level between the parameters PCL: posterior cruciate ligament; IKDC: International Knee Documentation Committee

Postop-preop		PCL index change
Lysholm score change	r	-0.119
	p	0.249
IKDC change	r	-0.098
	p	0.343

## Discussion

In the current study, postoperative PCL indexes in anatomical ACLR knees were significantly higher than preoperative values but lower than the healthy group. Despite a notable increase in postoperative functional scores, no statistically significant correlation was observed between this improvement and the increase in the PCL index. Despite the PCL index's relevance as an anatomical marker in post-ACLR evaluations, the current study's most noteworthy conclusion is that it does not seem to have any clear effect on functional outcomes.

The PCL acts as a primary constraint on posterior tibial translation [15]. The ACL serves as the principal static stabilizer versus tibial anterior translation [16] as well as contributes a minor role in internal rotational stability [17]. After ACL rupture, the disruption of ligament strain generates aberrant tibiofemoral incompatibility. The PCL index is formed by anterior tibial translation, which results in a decrease in strain and the bending of the PCL. Developing this indicator is linked to the concept that tendon reconstruction is related to tendon strain adjustment, joint alignment advancement, and functional recovery. Nevertheless, the correlation between the PCL index, structural reconstruction, and clinical importance in patients undergoing ACLR with HT allografts remains unclear.

Zampeli et al. found that the PCL indexes increased in patients who had ACLR but were low compared to the healthy group [10]. In the current study, the average PCL index measurement for the ACL-reconstructed category was 6.83. According to Nishimori et al. [9], the PCL index with unrestrained ACL grafts had average values of 5,283 and 5,608 for single- and double-bundle categories, respectively. In the current study, the increase in the average PCL index scores of the ACL-reconstructed sample was consistent with the results of other reports. When compared to the control participants, the average PCL index measurement for the reconstituted sample was aberrant, suggesting that there is still room for improvement in the pathological tibiofemoral position and the ability of anatomical ACLR to restore knee dynamics. The alteration in PCL morphology and the PCL index in ACL-deficient knees was thought to be caused by abnormal knee joint alignment [8,18].

The current study, like other investigations on the PCL index of ACL-reconstructed knees [5,8,9], revealed variations between ACL-reconstructed patients and matched controls. It is possibly due to anterior tibial subluxation, which occurs also in the operated limb after reconstruction, as well as to the reconstructed knee's internal rotation position with the ACL.

Since the IKDC and Lysholm surveys rely on translational exercises, their dependability is somewhat below satisfactory [19], and their distinction has decreased; hence, the reliability of these instruments in functional assessment may be impaired. This helps justify the absence of correlation, likely because the hamstring native tendons have an antagonising function that causes tension in the PCL medially [17,20]. Several reports propose that the hamstrings recover after two years of surgery [21,22]. However, the functional consequence of hamstring recovery is unknown. It can be hypothesised that hamstring damage, which reduces the rotational stability of the knee, is now the most common source of rotational instability, distorting the connection between the PCL index and clinical recovery. According to this view, Heijne et al. [23] identified a considerable difference in hamstring tension two years after surgery to encourage participants to undergo bone-patellar tendon-bone autograft (BPTB) reconstruction rather than HT reconstruction. Other studies have demonstrated that as compared to HT reconstruction, BPTB reconstruction can improve knee stability more efficiently [24,25] and yield better rotational stability [24,26]. It is probable that an association between the PCL index and clinical outcomes could not be detected in this study due to poor distinction of clinical results and rotational instabilities (caused by the HTs following extraction).

BPTB reconstruction was used in several research to assess the association between the PCL index and ACLR. When conducted with the BPTB autograft, restoring this index provided better control over rotational dynamics [10], but not anterior translational dynamics. Functional improvement was associated with rotational dynamics [27]. The hamstring muscles are vital in knee rotational stability, according to some publications [23-25]. In their research, Zampeli et al. [10,26] revealed that utilizing BPTB did not harm the HT or generate persistent rotational imbalance. The connection between rotational kinematics management and improved functional results highlights that the rotational part is indispensable in the biomechanics of ACL-reconstructed knees [27] and supports the fact that the HT provides rotational stability.

Pombo et al. [28] conducted an analysis on a sample of 50 patients, with 24 individuals in the operated group and 26 individuals in the control group. The findings of their study were comparable to the results of the present study. The current study differs from the previous one in that it examined a total of 191 patients, with 95 in the surgical group and 96 in the control group. This study is the most extensive examination in the literature on postoperative PCL index changes in a population of 95 operated patients. Moreover, a study conducted by Gong et al. in 2023 [29] revealed that the PCL index decreased as the disease progressed. Furthermore, it was observed that the PCL index in the chronic phase was significantly smaller than in the acute and subacute phases. However, there was no significant difference in the PCL index between the acute and subacute phases. In this investigation, we excluded measurements of patients in the acute phase and focused mostly on patients in the chronic phase. However, one drawback of our study is that we did not assess the PCL index values of the patients based on their respective phases.

Limitations and strengths

The data presented in the current study is subject to limitations, as is common with retrospective chart review studies. The data were not collected in a standardised manner using a prospective approach. Additionally, the reliability of the PCL index measurements performed by a radiologist was not assessed using intra-class correlation coefficients (ICC) by another radiologist. Additional limitations of the study include the exclusive utilization of a single type of surgery (anatomical ACLR using a single-bundle HT autograft), the absence of biomechanical analysis of the knee, the absence of inclusion of HT thickness in statistical analysis, and the relatively short duration of the follow-up period. Another limitation of the study is the lack of separate evaluation of PCL index values based on the specific injury phase (acute, subacute, chronic). A power analysis was not conducted prior to the commencement of the study to identify the optimal sample size. However, the current study seems the most extensive investigation in the existing body of literature that analyses PCL index values after ACLR surgery.

## Conclusions

The PCL index is a usable morphological metric during the follow-up period of ACLR using HT autograft; however, it is poorly linked to functional outcomes. With the largest sample size to date, the current study provides the most thorough evaluation of PCL index alterations after ACLR with HT autograft. Further studies with longer follow-up periods and biomechanical examinations of the association between the PCL index values and clinical results are needed.
